# Counting cytoplasmic incompatibility factor mRNA using digital droplet PCR

**DOI:** 10.1128/spectrum.02347-25

**Published:** 2026-02-12

**Authors:** Lore Van Vlaenderen, William R. Conner, J. Dylan Shropshire

**Affiliations:** 1 Department of Biological Sciences, Lehigh University 1687, Bethlehem, Pennsylvania, USA; 2 Division of Biological Sciences, University of Montana 307078, Missoula, Montana, USA; Brigham Young University, Provo, Utah, USA

**Keywords:** *Drosophila*, *Wolbachia*, RT-ddPCR, symbiosis, cytoplasmic incompatibility

## Abstract

*Wolbachia* bacteria inhabit over half of all insect species and often spread through host populations via efficient maternal transmission and cytoplasmic incompatibility (CI), killing aposymbiotic embryos when fertilized by symbiotic males. *Wolbachia*’s *cifB* gene triggers CI in males, while *cifA*, expressed in females, rescues embryos from CI-induced lethality. In some systems, *cifA* also contributes to CI induction. CI strength—the percentage of embryos that die from CI—is a key determinant of *Wolbachia*’s prevalence in host populations, and *cifB*-mRNA levels in testes generally correlate with CI strength. Yet, *cifB*’s rarity can hamper precise quantification, necessitating tissue pooling for reverse transcription quantitative PCR (RT-qPCR) to achieve reliable measurements, obscuring variation at the level of individual insect tissues. Here, we present four RT digital droplet PCR (RT-ddPCR) assays to count rare *cifA* and *cifB* mRNA from *w*Mel *Wolbachia* in *Drosophila melanogaster*. These assays count *cif* transcripts alongside a synthetic spike-in RNA or a *D. melanogaster* reference gene to normalize for technical or biological variation. These assays have a limit of detection of about one *cifA* and three *cifB* copies per reaction. We expect these methods to be useful for mosquito-control programs that use *w*Mel to block the spread of pathogens from *Aedes aegypti* to humans. Moreover, the oligos were designed with homology to *cifA* and *cifB* sequences from at least 34 *Wolbachia* strains, suggesting potential utility beyond *w*Mel. These methods will allow researchers to measure *cif*-mRNA levels from individual insect tissues, enabling efforts to pair molecular and phenotypic data at unprecedented resolutions.

**IMPORTANCE:**

*Wolbachia*, a maternally transmitted bacterium, is found in over half of all insect species. Its ability to induce cytoplasmic incompatibility (CI), which prevents *Wolbachia*-free eggs from hatching, significantly contributes to its high prevalence in host populations. Public health experts use CI to spread pathogen-blocking *Wolbachia* through mosquito populations, thereby controlling pathogen spread. CI is often weak, resulting in few egg deaths and consequently slowing *Wolbachia*’s spread. We recently discovered that weak CI often correlates with low CI factor B (*cifB*) mRNA levels. However, our understanding of CI-strength variation remains limited because *cifB* is transcribed at low levels, making it challenging to measure in individual insects. Here, we report four RT-ddPCR assays to overcome this challenge. These assays offer high sensitivity for rare targets and maintain accuracy and precision across a wide dynamic range. We expect these tools will enhance efforts to understand CI-strength variation in both natural and applied populations.

## INTRODUCTION

Many insects host intracellular bacteria that mothers pass to their offspring (1, 2). Among these microbes, the Alphaproteobacterium *Wolbachia* (3) is one of the most common, found in over half of all insect species (4), and often widespread within host populations (5). *Wolbachia*’s high prevalence within host populations largely stems from its ability to cause cytoplasmic incompatibility (CI) (6, 7). CI occurs when a symbiotic male mates with an aposymbiotic female, killing the resulting embryos (8). Conversely, embryos from symbiotic females resist CI, giving *Wolbachia*-bearing offspring a selective advantage. Two *Wolbachia* genes orchestrate CI: *cifB* causes CI when expressed in testes, and *cifA* rescues CI when expressed in ovaries (9–15). In some systems, both *cifA* and *cifB* must be co-expressed in males to induce CI, for unknown reasons (11). The precise molecular and cellular mechanisms of CI are reviewed elsewhere (7). Beyond its ecological role, public health experts use CI to spread pathogen-blocking *Wolbachia* strains through *Aedes aegypti* mosquito populations, thereby protecting humans from diseases such as dengue and Zika (16–19).

Strong CI is characterized by high embryo mortality and is crucial for *Wolbachia*’s spread to high frequencies in both natural and applied populations (6, 20, 21). However, CI strength can vary significantly with host and *Wolbachia* genetics (10, 21–24), male age (25–27), mating frequency (25, 28), diet (29, 30), temperature (31, 32), and other factors (reviewed in reference 7). Although we still lack a complete understanding of what causes variation in CI strength, CI tends to be stronger when *cifB*-mRNA levels are higher. This correlation is observed when comparing *Wolbachia* strains across *Drosophila* species and when CI weakens as *w*Ri-bearing *D. simulans* males get older (24, 27).

Reverse transcriptase quantitative PCR (RT-qPCR) is traditionally used to measure *cifA*- and *cifB*-mRNA levels (24, 27). This method requires purifying total RNA, synthesizing complementary DNA (cDNA) through reverse transcription, and then performing PCR with the inclusion of a fluorescent DNA-binding probe or an intercalating dye, such as SYBR Green, to monitor amplicon accumulation. Each PCR cycle doubles the target amplicon abundance, which in turn doubles the fluorescent signal. Researchers infer the initial quantity of target cDNA by identifying the cycle at which its fluorescent signal crosses a predetermined threshold. While *cifA* mRNA is typically abundant and readily measured by RT-qPCR, *cifB* transcripts can be rare (33, 34). Detecting *cifB* commonly requires more than 30 amplification cycles to reach the detection threshold, even when cDNA is derived from samples containing tissues pooled from 15 or more individuals (24, 27). These expression dynamics hinder efforts to correlate molecular data with phenotypic outcomes in individual insects and complicate studies in systems where *cifB* transcripts are especially rare.

Compared to RT-qPCR, reverse transcriptase digital droplet PCR (RT-ddPCR) offers enhanced sensitivity, accuracy, and precision for absolute quantification (35). While RT-ddPCR shares initial steps with RT-qPCR—including RNA extraction, cDNA synthesis, and PCR setup—it differs in several key ways. First, each reaction is partitioned into thousands of nanoliter-sized droplets. Second, PCR is performed to completion on each droplet. Third, a microfluidic device counts droplets and uses fluorescence thresholds to classify each as positive or negative for the target. Finally, the absolute number of target molecules is calculated using Poisson statistics, which corrects for the probability of a droplet containing more than one target molecule. Endpoint detection eliminates the assumption that each PCR is 100% efficient and reduces sensitivity to inhibitors commonly introduced during RNA purification. Additionally, the large number of droplets per reaction (10,000–20,000 in the QX200 system used here) provides robust statistical power, enabling detection of as few as one target molecule with 95% confidence (36–38).

In *w*Mel *Wolbachia*, the strain naturally inhabiting *D. melanogaster*, both *cifA* and *cifB* are necessary for CI induction (11, 12). Here, we present optimized methods for the extraction, purification, and processing of RNA from *D. melanogaster* testes to count rare *cifA* and *cifB* mRNA of *w*Mel *Wolbachia* using two-step RT-ddPCR. We first optimized a phase-separation RNA extraction protocol that consistently yields ~52.5 ng RNA per pair of testes, with extraction efficiency remaining stable across samples containing 1 to 20 testis pairs. We then validated DNase treatment protocols to completely eliminate genomic DNA contamination, which is essential for distinguishing mRNA-derived signals from genomic DNA in RT-ddPCR assays. For each *cif* gene, we developed two duplex RT-ddPCR assays: *cif*/spike and *cif*/*β-Spec*. The *cif*/spike assays account for technical variation by measuring *cif* mRNA levels against a spike-in RNA control, while the *cif*/*β-Spec* assays control for biological variation by normalizing against the *D. melanogaster* reference gene *β Spectrin*. We validated these assays using pooled RNA and serial dilutions to isolate technical performance from biological variation, establishing accuracy across a broad dynamic range, precision between technical replicates, and sensitivity down to 1 to 3 *cifA* and *cifB* copies per 20 µL reaction. This sensitivity, combined with efficient RNA extraction from individual tissues, enables individual-level *cif*-mRNA quantification even at low expression levels. Designed with homology to *cifA* and *cifB* variants in at least 34 *Wolbachia* strains, we expect these assays to be applicable beyond *w*Mel in *D. melanogaster* and *Ae. aegypti*, where it is used to control mosquito-borne disease. While ddPCR has been used to measure *Wolbachia* abundance in multiple studies (39–43), to our knowledge, this is the first application of RT-ddPCR to count *Wolbachia* mRNA.

## RESULTS

### Optimizing RNA purification for low-biomass samples

**Fig 1:**
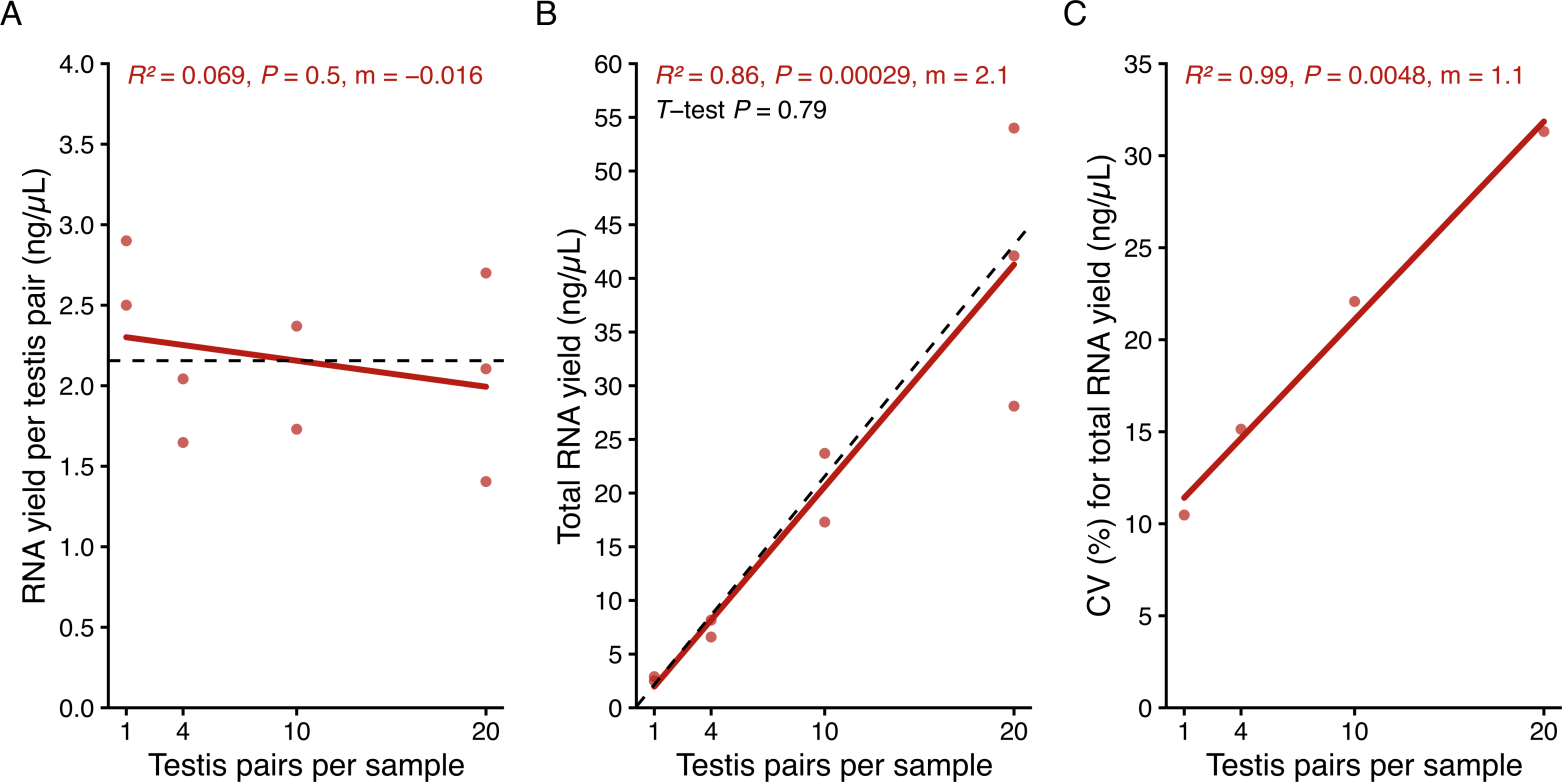
Phase-separation RNA extraction consistently yields about 2.1 ng/µL of RNA per pair of testes. (**A**) RNA yield was normalized to the number of testis pairs in each sample, revealing consistent per-pair recovery (~2.1 ng/µL) regardless of the number of testis pairs pooled per sample. (**B**) Total RNA concentration significantly increases with the number of testes per sample. The dashed black line indicates the expected linear relationship between total RNA yield and the number of testes, calculated using the average RNA yield per pair of testes as the slope. We used a one-sample *t*-test to compare the observed and expected slopes. (**C**) Technical variability in sample-to-sample RNA yield, as measured by the coefficient of variation (CV), is significantly higher in sample groups containing more testes. (**A–C**) We quantified RNA concentrations using a Qubit 4 Fluorometer with the Qubit RNA High Sensitivity Kit. Each plot displays Pearson’s correlation coefficient, the corresponding *P* value, and the regression line’s slope (M). Sample size: (**A and B**) nine independent RNA extractions spanning four testis-pooling groups (1, 4, 10, or 20 pairs per extraction). (**C**) Four CV values (one per pooling group, each calculated from two to three biological replicates per group). Raw data are available in Data S1: https://datadryad.org/dataset/doi:10.5061/dryad.0k6djhbd8.

To enable sensitive RT-ddPCR assays for rare *cifA* and *cifB* mRNA in individual insects, we first developed and validated an RNA extraction protocol optimized for low-biomass samples. Due to its documented yields being equivalent to or exceeding column-based extraction methods when normalized by input biomass, we decided to extract and purify RNA through phase separation (44, 45) (see Materials and Methods for details). Adding an extra ethanol reprecipitation step significantly improves RNA purity by reducing both protein (average A260/A280 improved from 1.87 to 1.94; paired *t*-test *P* = 0.041, *N =* 9) and chemical contamination (average A260/A230 improved from 0.93 to 1.45; paired *t*-test *P* = 9.8e−3, *N =* 9) without significantly impacting RNA yield (paired *t*-test *P* = 0.44, *N =* 9) (Data S1 available at https://datadryad.org/dataset/doi:10.5061/dryad.0k6djhbd8). We tested this protocol’s accuracy and precision across samples with different biomass by extracting RNA from 1, 4, 10, and 20 pairs of *w*Mel-bearing *D. melanogaster* testes. RNA yield per pair of testes is consistent regardless of the number of testes in the sample (*R^2^* = 0.069, *P* = 0.5, *N =* 9), averaging 2.1 ng/μL per pair of testes in 25 μL of low EDTA buffer (52.5 ng total; Fig. 1A). Consequently, the total RNA concentration significantly increases with the number of testes (Pearson’s *R^2^* = 0.86, *P* = 2.9e−4, *N =* 9; Fig. 1B). The observed slope of this relationship is consistent with the average yield per testis pair (one sample *t*-test *P* = 0.79, *N =* 9), supporting accurate recovery across biomass levels. However, sample-to-sample variation in RNA yield increases with tissue abundance (Pearson’s *R^2^* = 0.99, *P* = 4.8e−3, *N =* 4; Fig. 1C). We attribute this to compounding technical imprecision: while the extraction protocol is identical for all samples, small variations at each step (e.g., homogenization efficiency, phase recovery, pellet washes) produce proportionally larger effects on yield when more tissue is processed. These findings demonstrate that this RNA extraction and purification protocol yields about 52.5 ng of RNA per pair of testes and is suitable for samples containing 1 to 20 pairs of testes. The choice between individual and pooled samples should be based on experimental goals. Spike-in controls (described below) can help account for variation in purification efficiency and may be particularly useful when using pooled samples, where sample-to-sample yields are more variable.

### Evaluating DNase treatment kits

**Fig 2:**
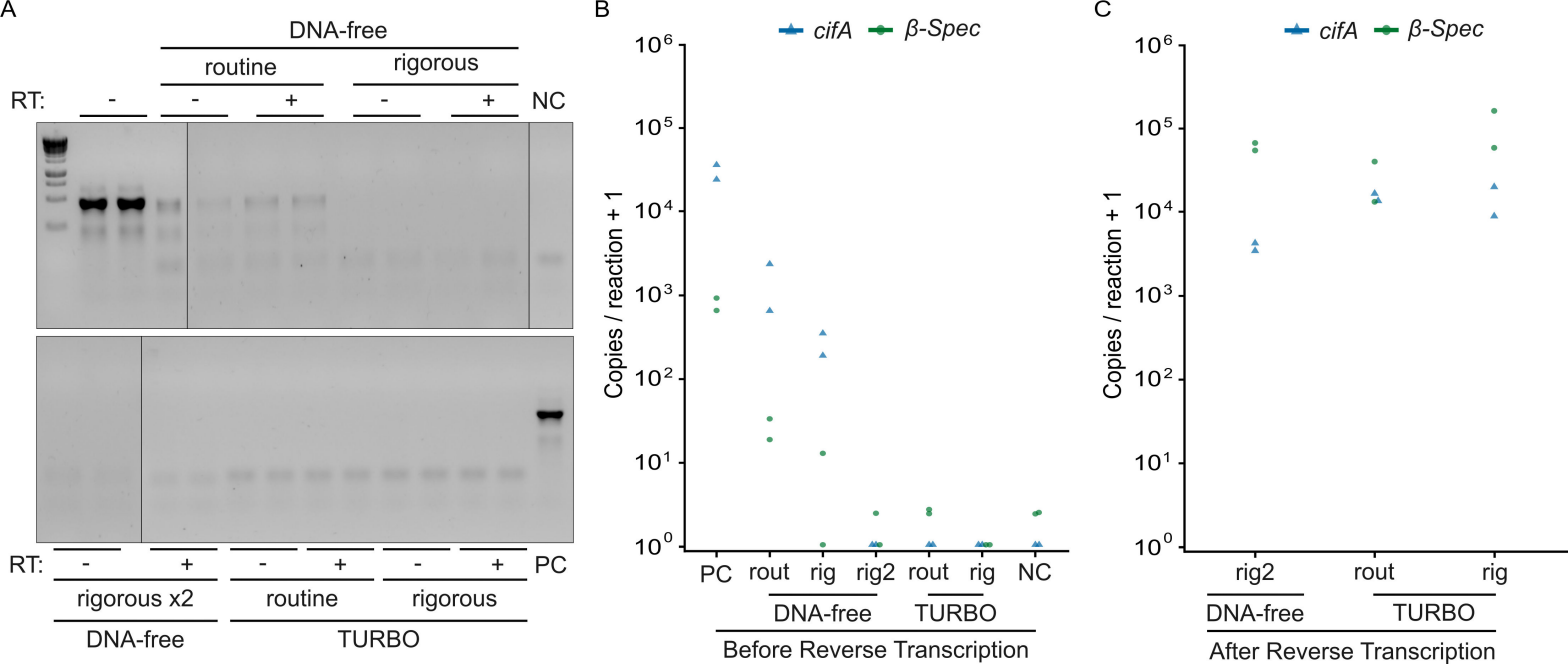
Treatment with TURBO DNase can completely remove contaminating DNA from RNA samples. (**A**) We could not detect *D. melanogaster* 28S rDNA amplified by PCR and gel electrophoresis in RNA treated with either the rigorous DNA-free or any TURBO DNase protocols. In contrast, the DNA-free routine protocol showed a visible 28S-associated amplicon. Black vertical lines indicate locations where the gel was cropped for display. (**B**) All DNase treatment protocols reduced *cifA*- and *β-Spec*-gDNA copy numbers compared to non-DNase-treated controls. We detected no *cifA* gDNA in the DNA-free rigorous ×2, TURBO routine, and TURBO rigorous protocols. Only the TURBO rigorous protocol completely removed *β-Spec* gDNA. (**C**) Among the protocols that achieved complete *cifA* gDNA removal, *cifA* and *β-Spec* RNA concentrations after reverse transcription are similar. Sample size: (**A–C**) two independent RNA extractions per DNase treatment condition. Raw gel images and data are available in Data S2 and S3: https://datadryad.org/dataset/doi:10.5061/dryad.0k6djhbd8.

Minimizing genomic DNA (gDNA) carryover from RNA purifications is crucial, especially when the goal is to identify and count rare transcripts from low-biomass samples. To establish a suitable methodology, we evaluated gDNA leftovers in RNA samples treated with five different DNase protocols (see Materials and Methods for details). After DNase treatment, we assessed gDNA presence using two approaches. First, we amplified a non-transcribed region of *D. melanogaster*’s 28S rDNA using standard PCR and visualized the amplicon via gel electrophoresis. We observe a 28S-associated amplicon when using the DNA-free routine kit. All other protocols yield no detectable DNA (Fig. 2A). Second, we used a *cifA*/*β-Spec* RT-ddPCR assay (described below) to quantify *cifA* (*Wolbachia*-encoded) and *β-Spec* (host-encoded) gDNA contamination. In non-DNase-treated RNA samples, we detected 32,141 *cifA* gDNA and 812 *β-Spec* gDNA copies per reaction, confirming high levels of gDNA contamination. All five DNase treatment protocols significantly reduced gDNA contamination: *cifA* gDNA by at least 95.15% and *β-Spec* gDNA by at least 96.93% (Fig. 2B). However, residual gDNA remained detectable with the DNA-free routine (1,558 *cifA* and 25 *β-Spec* gDNA copies per reaction) and rigorous (548 *cifA* and 6 *β-Spec* gDNA copies per reaction) protocols, indicating incomplete digestion. In contrast, we detected 0 *cifA* gDNA copies per reaction with the DNA-free rigorous ×2 protocol and both TURBO protocols. Only the TURBO rigorous protocol completely eliminated *β-Spec* gDNA contamination. Among the three protocols that completely removed *cifA* gDNA, *cifA* and *β-Spec* cDNA concentrations were similar after reverse transcription (Fig. 2C), indicating that complete gDNA removal did not compromise RNA integrity or RT efficiency. Therefore, we conclude that treating purified RNA from testes extracts with the TURBO rigorous protocol is sufficient to digest all gDNA.

### Developing generalizable *cif* RT-ddPCR assays

**Fig 3:**
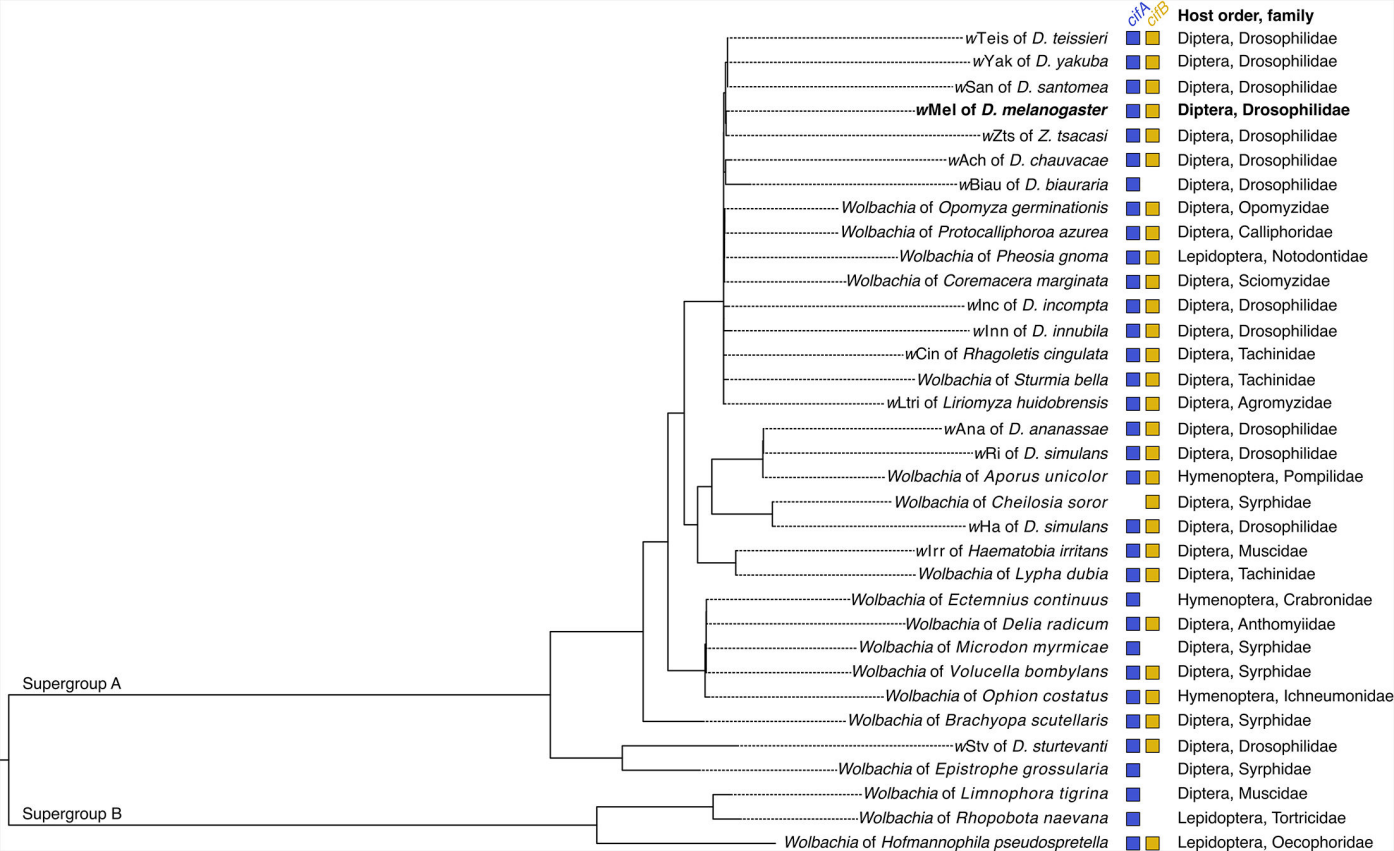
*cifA* and *cifB* oligos are homologous to *cif* sequences in supergroup A and B *Wolbachia* strains. Phylogram of 34 *Wolbachia* strains based on 149 full-length single-copy genes (117,282 bp). We used Primer-BLAST to identify oligo homology across *Wolbachia* genomes. Strains are included if forward primer, reverse primer, and probe sequences show 100% homology to *cifA*, *cifB*, or both genes. *Wolbachia* from *Limnophora tigrina*, *Rhopobota naevana*, and *Hofmannophila pseudospretella* are supergroup B; all others are supergroup A. Nodes with posterior probabilities <0.95 are collapsed into polytomies. Colored blocks to the right indicate perfect oligo homology to *cifA* (blue; left) or *cifB* (yellow; right) target sequences in each strain’s genome. In this study, the *cifA* and *cifB* oligos were only validated for the *w*Mel strain (bold text). While oligos are homologous to *cif* sequences in other strains, additional validation is required.

To design primers and probes for the *cifA* and *cifB* mRNA of the *w*Mel *Wolbachia* strain from *D. melanogaster*, we used an iterative approach involving multiple sequence alignments, consensus sequence generation, and primer design tools (see Materials and Methods for details). We validated the primers against the NCBI nr database using NCBI Primer-BLAST. This confirmed no off-target binding to humans (taxid: 9606), *Drosophila* (taxid: 7215), and *Wolbachia* (taxid: 953). Primer-BLAST analysis identified homology across diverse *Wolbachia* genomes: *cifA* oligos show homology to 39 sequences and *cifB* oligos to 34 sequences, spanning 31 supergroup A and 3 supergroup B strains (Fig. 3; Data S4 and S5 available at https://datadryad.org/dataset/doi:10.5061/dryad.0k6djhbd8). This broad homology includes *Wolbachia* from 12 *Drosophila* species, multiple other Diptera families (e.g., Syrphidae, Tachinidae, Muscidae), and representatives from Hymenoptera and Lepidoptera.

### Evaluating RT-ddPCR droplet differentiation

**Fig 4:**
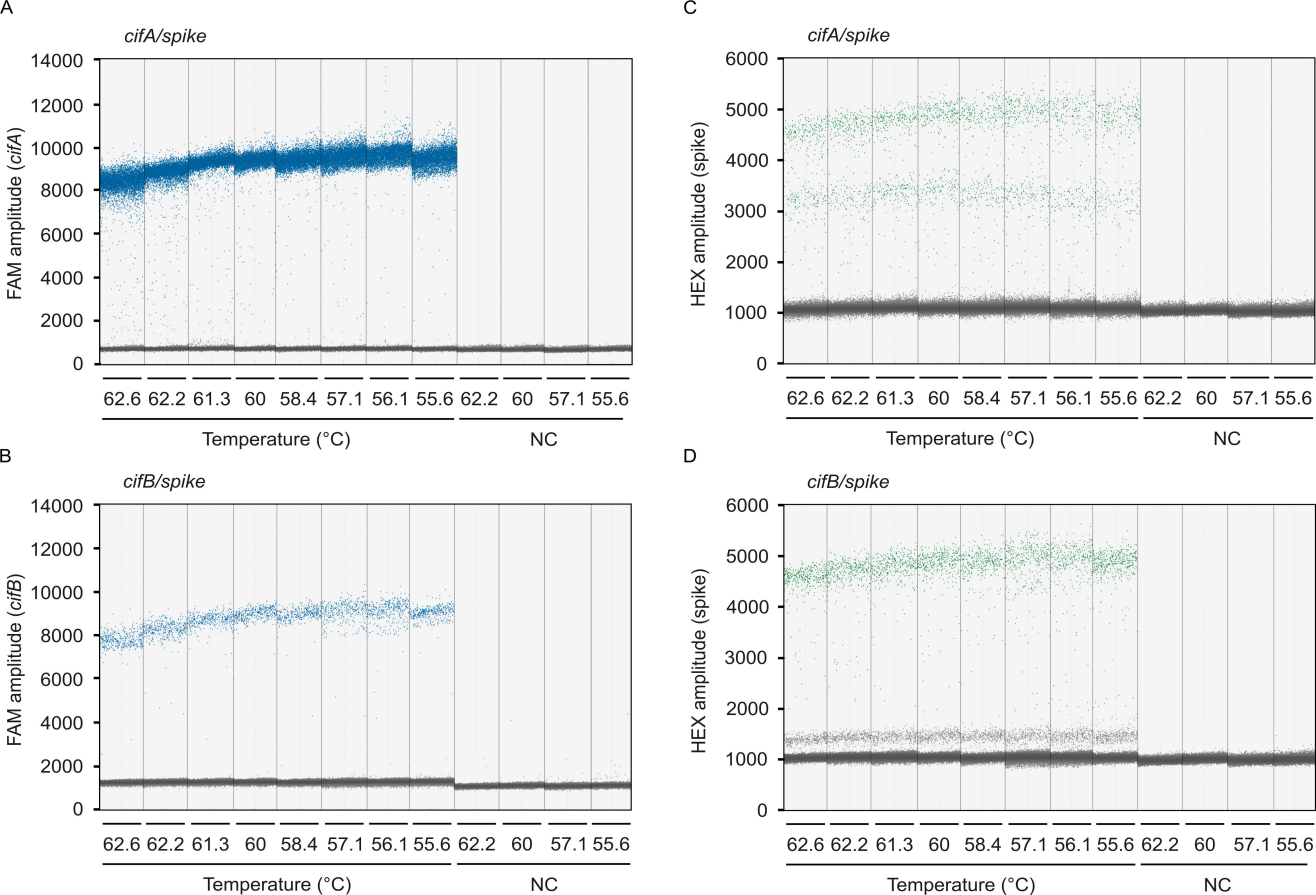
*cif*/spike RT-ddPCR droplet discrimination is robust across annealing temperatures. *cifA* and *cifB* detection on the FAM channel is consistent across a range of annealing temperatures (62.6°C to 55.6°C) in the (**A**) *cifA*/spike and (**B**) *cifB*/spike assays. Synthetic spike-in detection on the HEX channel is consistent across annealing temperatures in the (**C**) *cifA*/spike and (**D**) *cifB*/spike assays. (**A–D**) Each data point represents an individual droplet. Droplets with high fluorescence amplitude (blue on FAM channel; green on HEX channel) contain the target template, while low-amplitude droplets (gray) lack the target. Clear separation between high- and low-amplitude populations indicates robust assay performance. Vertical dotted lines delineate the results from 20 μL RT-ddPCRs, each containing 2 μL of cDNA template derived from RNA purified from 20 pairs of testes. Each reaction yielded no fewer than 10,000 droplets. NC, no-template control. Sample size: (**A–D**) eight experimental RT-ddPCRs per plot (one reaction per annealing temperature), all derived from a single cDNA sample. Raw data are available in Data S6 and S7: https://datadryad.org/dataset/doi:10.5061/dryad.0k6djhbd8.

**Fig 5:**
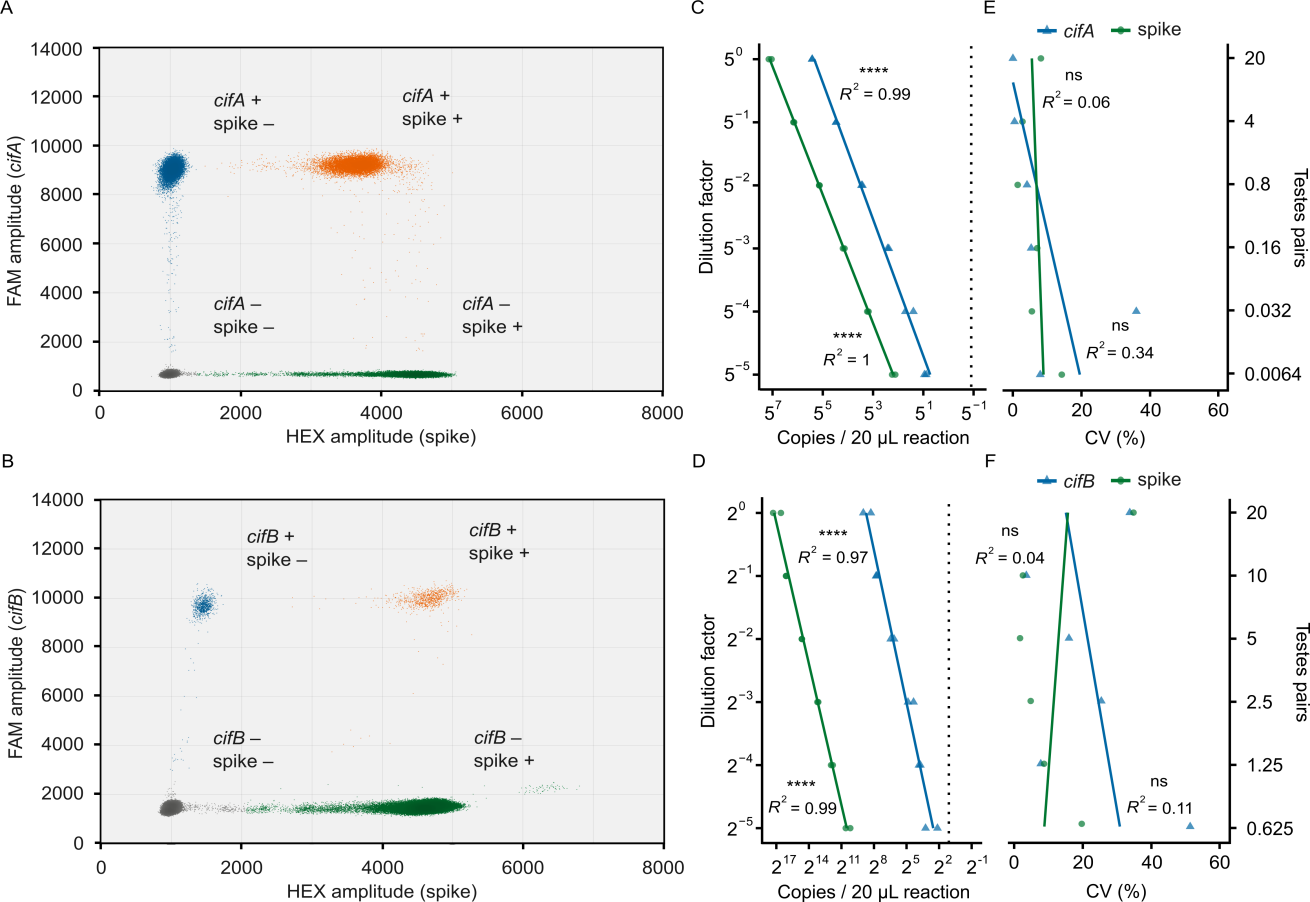
The *cif*/spike RT-ddPCR assays are accurate and precise. Fluorescence amplitudes of droplets from (**A**) *cifA*/spike and (**B**) *cifB*/spike RT-ddPCRs. Each point represents an individual droplet positioned according to its fluorescence intensity on the HEX (*x*-axis) and FAM (*y*-axis) channels. Droplets are colored to indicate their content: gray (no targets), blue (*cif* only), green (spike only), and orange (both targets). Distinct clustering into four populations demonstrates that the assay can simultaneously quantify both targets without interference. We calculated target concentrations from both single- and double-positive droplets. Relationship between dilution factor and target copy number in (**C**) *cifA*/spike and (**D**) *cifB*/spike RT-ddPCR assays. Vertical dotted lines indicate the *cif* limits of detection, based on the upper limit of the 95% CI from no-template controls. Strong linear correlations confirm that both assays are accurate across broad dynamic ranges above the limit of detection. The relationship between dilution factor and coefficients of variation (CV) that measure technical precision in the (**E**) *cifA*/spike and (**F**) *cifB*/spike assays. Non-significant correlations indicate that precision remains consistent across the dilution series. (**A–F**) All 20 µL RT-ddPCRs contained 2 µL of cDNA template derived from RNA extracted from 20 pairs of testes, or dilutions thereof. Each reaction yielded no fewer than 10,000 droplets. Statistical tests are (**C–F**) Pearson’s product–moment correlations. Statistical significance is denoted as: *P* > 0.05 (ns), *P* ≤ 0.05 (*), *P* ≤ 0.01 (**), *P* ≤ 0.001 (***), *P* ≤ 0.0001 (****). Sample size: (**A and B**) 12 RT-ddPCRs per assay (pooled across the six dilution levels shown in C and D); all derived from a single cDNA sample; (**C and D**) 12 ddPCRs per assay (two technical replicates per dilution level), all derived from a single cDNA sample; (**E and F**) six CV values per assay and target (one per dilution level, each calculated from two technical replicates). Raw data are available in Data S8 and S9: https://datadryad.org/dataset/doi:10.5061/dryad.0k6djhbd8.

We developed duplex RT-ddPCR assays pairing the newly designed *cif* oligos with a commercially available RNA spike-in control (TATAA Biocenter, RS25SI). These assays simultaneously count *cifA* or *cifB* (FAM channel) and a synthetic spike-in RNA (HEX channel) molecule. We used 2 µL of cDNA from samples extracted from 20 pairs of testes as the template in each 20 µL reaction. Both assays produced two clear FAM populations, indicating the presence or absence of *cif* targets (Fig. 4A and B). On the HEX channel, however, three droplet clusters appeared in both assays (Fig. 4C and D). Two-dimensional analysis of FAM and HEX fluorescence revealed that intermediate clusters represent different droplet populations in each assay: in the *cifA*/spike assay, the intermediate cluster contains both *cifA* and spike-in RNA, while in the *cifB*/spike assay, it contains only *cifB* (Fig. 5A and B). We hypothesize that in the *cifA*/spike assay, reaction competition between targets reduces spike-in RNA amplification in double-positive droplets, thereby decreasing spike-in RNA copy number per droplet and HEX fluorescence. Conversely, in the *cifB*/spike assay, the intermediate HEX fluorescence in *cifB*-positive droplets likely results from spectral overlap, where high FAM fluorescence contributes to the HEX channel signal. Thus, despite the appearance of intermediate clusters, the assays reliably distinguish droplets containing no template, a single template (*cif* or spike-in RNA), or both templates based on their distinct fluorescence profiles.

### Testing RT-ddPCR efficiency across annealing temperatures

To determine the optimal annealing temperature for the *cif*/spike RT-ddPCR assays, we tested RT-ddPCR performance across a temperature gradient ranging from 55.6°C to 62.6°C using 2 μL of cDNA derived from 20 pairs of testes per 20 μL reaction. The optimal temperature was defined as that which produced the largest amplitude difference between positive and negative droplets. Fluorescence amplitudes of negative droplets are consistent across temperature treatments for *cifA* (FAM; *${\bar{x}}_{\min}$* = 691.89, ${\bar{x}}_{\max}$ = 712.72, *N =* 8; Fig. 4A), *cifB* (FAM; ${\bar{x}}_{\min}$ = 1,230.30, ${\bar{x}}_{\max}$ = 1,273.32, *N =* 8; Fig. 4B), and spike (HEX; *${\bar{x}}_{\min}$* = 1,032.39, ${\bar{x}}_{\max}$ = 1,090.80, *N =* 8; Fig. 4C and D). Conversely, positive droplet signal amplitude depends on temperature. We detect the highest amplitude for *cifA* at 56.1°C ($\bar{x}$ = 9,603.08; Fig. 4A), *cifB* at 56.1°C ($\bar{x}$ = 9,069.59; Fig. 4B), and spike at 55.6°C (*cifA*/spike; $\bar{x}$ = 4,283.91; Fig. 4C) and 58.4°C (*cifB*/spike; $\bar{x}$ = 4,808; Fig. 4D). However, the separation between positive and negative droplet clusters is unambiguous at all tested temperatures. Based on this performance, we selected 60°C as the standard annealing temperature for all subsequent experiments.

### Validating *cif*/spike RT-ddPCR assays

To assess the accuracy of the *cif*/spike assays, we tested each assay against a cDNA dilution series. We derived the cDNA from RNA purified from three samples, each containing 20 pairs of *Drosophila* testes. We used a 1:5 dilution factor for the *cifA*/spike assays and a 1:2 factor for the *cifB*/spike assays. We performed RT-ddPCR across these dilutions using 2 µL of cDNA per 20 µL reaction and calculated target concentrations from both single-positive and double-positive droplets. For these assays to be practical for analyzing single testes pairs, we needed to reliably detect *cifA* at the 5^−2^ dilution (equivalent to 0.8 testes pairs) and *cifB* at the 2^−5^ dilution (equivalent to 0.625 testes pairs). We detect 374.86 *cifA* copies/reaction at the 5^−2^ dilution (Fig. 5C) and 8.20 *cifB* copies/reaction at the 2^−5^ dilution (Fig. 5D). These values are above the limit of detection, defined by the upper limit of the 95% confidence interval (CI) for the no-template controls (0.54 *cifA* and 2.59 *cifB* copies/reaction, respectively). Furthermore, we observe a strong, positive linear relationship between the dilution level and the measured abundance for both the *cifA*/spike assay (*cifA* Pearson’s *R^2^* = 0.99, *P* = 6.88e−12, *N =* 12; spike Pearson’s *R^2^* = 1, *P* = 2.21e−17, *N =* 12; Fig. 5C) and the *cifB*/spike assay (*cifB* Pearson’s *R^2^* = 0.97, *P* = 5.16e−9, *N =* 12; spike Pearson’s *R^2^* = 0.99, *P* = 3.23e−11, *N =* 12; Fig. 5D). Collectively, these data confirm that both *cifA*/spike and *cifB*/spike assays are suitable for counting mRNA from individual testes pairs and accurately measuring the abundance of their respective targets across a broad range of concentrations.

We evaluated assay technical precision by calculating the coefficient of variation between technical replicates (two per reaction type) at each dilution factor. As expected (see Discussion), precision declined as target concentrations decreased for *cifA* and *cifB*, although not significantly. For instance, the coefficient of variation for *cifA* in the *cifA*/spike assay increased from 0.03% at the 5^0^ dilution to 7.98% at the 5^−5^ dilution (Pearson’s *R^2^* = 0.338, *P* = 0.226, *N =* 6; Fig. 5C). Similarly, the coefficient of variation for *cifB* in the *cifB*/spike assay rose from 12.29% at the 2^0^ dilution to 33.14% at the 2^−5^ dilution (Pearson’s *R^2^* = 0.109, *P* = 0.522, *N =* 6; Fig. 5D). We observed similar patterns for the spike-in sequence in both assays (*cifA*/spike Pearson’s *R^2^* = 0.0615, *P* = 0.635, *N =* 6; *cifB*/spike Pearson’s *R^2^* = 0.0387, *P* = 0.709, *N =* 6; Fig. 5C and D). These data demonstrate that the *cif*/spike assays exhibit good precision, albeit with lower precision as targets are made rarer.

### Validating *cif*/*β-Spec* RT-ddPCR assays

**Fig 6:**
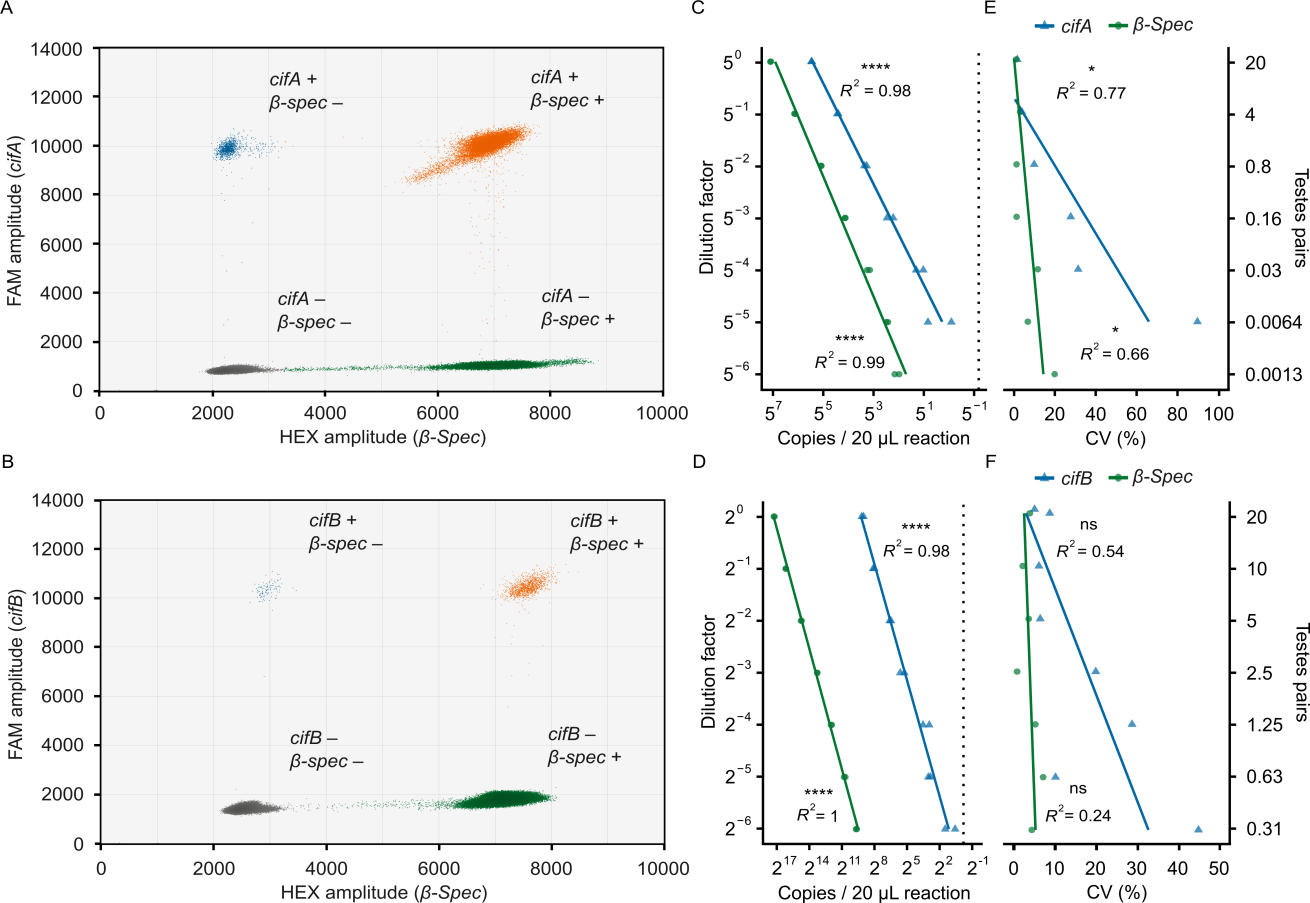
The *cif*/*β-Spec* RT-ddPCR assays are accurate and precise. Fluorescence amplitudes of droplets from (**A**) *cifA*/*β-Spec* and (**B**) *cifB*/*β-Spec* RT-ddPCRs. Each point represents an individual droplet positioned according to its fluorescence intensity on the HEX (*x*-axis) and FAM (*y*-axis) channels. Droplets are colored to indicate their content: gray (no targets), blue (*cif* only), green (*β-Spec* only), and orange (both targets). Distinct clustering into four populations demonstrates that the assay can simultaneously quantify both targets without interference. We calculated target concentrations from both single- and double-positive droplets. Relationship between dilution factor and target copy number in (**C**) *cifA*/*β-Spec* and (**D**) *cifB*/*β-Spec* RT-ddPCR assays. Vertical dotted lines indicate the *cif* limits of detection, based on the upper limit of the 95% CI from no-template controls. Strong linear correlations confirm that both assays are accurate across broad dynamic ranges above the limit of detection. The relationship between dilution factor and coefficients of variation (CV) that measure technical precision in the (**E**) *cifA*/*β-Spec* and (**F**) *cifB*/*β-Spec* assays. The *cifA*/*β-Spec* assay demonstrates lower technical precision at low concentrations, while precision in the *cifB*/*β-Spec* assay does not significantly vary across the dilution series. (**A–F**) All 20 µL RT-ddPCRs contained 2 µL of cDNA template derived from RNA extracted from 20 pairs of testes, or dilutions thereof. Each reaction yielded no fewer than 10,000 droplets. Statistical tests are (**C–F**) Pearson’s product–moment correlations. Statistical significance is denoted as: *P* > 0.05 (ns), *P* ≤ 0.05 (*), *P* ≤ 0.01 (**), *P* ≤ 0.001 (***), *P* ≤ 0.0001 (****). Sample size: (**A and B**) 14 RT-ddPCRs per assay (pooled across the seven dilution levels shown in panels C and D), all derived from a single cDNA sample; (**C and D**) 14 ddPCRs per assay (two technical replicates per dilution level), all derived from a single cDNA sample; (**E and F**) seven CV values per assay and target (one per dilution level, each calculated from two technical replicates). Raw data are available in Data S10 and S11: https://datadryad.org/dataset/doi:10.5061/dryad.0k6djhbd8.

In addition to the *cif*/spike RT-ddPCR assays, we developed duplex RT-ddPCR assays to simultaneously count *cifA* or *cifB* and a *D. melanogaster* reference gene, *β-Spec*. Similar to the *cif*/spike assays, representative reactions for both the *cifA*/*β-Spec* (Fig. 6A) and *cifB*/*β-Spec* (Fig. 6B) assays demonstrate clear separation of droplet populations, enabling unambiguous quantification of both targets. We assessed the accuracy of the *cif*/*β-Spec* assays using the same methodology as the *cif*/spike assays. In brief, we prepared serial cDNA dilutions from mRNA derived from 20 pairs of testes (a 1:5 dilution factor for *cifA*/*β-Spec* and 1:2 for *cifB*/*β-Spec*) and performed RT-ddPCR using 2 µL of cDNA per 20 µL reaction. For these assays to be practical for counting *cif* mRNA from individual testes pairs, they needed to reliably detect *cifA* at the 5^−2^ dilution and *cifB* at the 2^−5^ dilution. We detect 356.79 *cifA* copies/reaction (Fig. 6C) and 8.13 *cifB* copies/reaction (Fig. 6D) at these respective concentrations. These values are above the limits of detection, defined by the upper limit of the 95% CI of the no-template controls (0.59 *cifA* and 0.87 *cifB* copies/reaction). Consistent with the *cif*/spike assay results, dilution factor and the measured abundance for both the *cifA*/*β-Spec* assay (*cifA* Pearson’s *R^2^* = 0.985, *P* = 1.94e−10, *N =* 14; *β-Spec* Pearson’s *R^2^* = 0.986, *P* = 1.59e−12, *N =* 14; Fig. 6C) and the *cifB*/*β-Spec* assay (*cifB* Pearson’s *R^2^* = 0.98, *P* = 1.52e−11, *N =* 14; *β-Spec* Pearson’s *R^2^* = 0.998, *P* = 2.91e−17, *N =* 14; Fig. 6D) are strongly correlated. Collectively, these data confirm that, similar to the *cif*/spike assays, both the *cifA*/*β-Spec* and *cifB*/*β-Spec* assays are well-suited for detecting mRNA from individual testes pairs and accurately measuring target abundance across a wide dynamic range.

Next, we assessed the technical precision of the *cif*/*β-Spec* assays by calculating the coefficient of variation between technical replicates (two per reaction type) for each dilution. The coefficients of variation for *cifA* significantly increase from 1.64% at the 5^0^ dilution to 89.74% at the 5^−6^ dilution (Pearson’s *R²* = 0.771, *P* = 0.0215, *N =* 7; Fig. 6E). Similarly, the coefficients of variation for *cifB* increase, though not significantly, from 8.01% at the 2^0^ dilution to 43.57% at the 2^−5^ dilution (Pearson’s *R²* = 0.54, *P* = 0.0584, *N =* 7; *β-Spec* Pearson’s *R²* = 0.24, *P* = 0.266, *N =* 7; Fig. 6F). For *β-Spec*, we observed a significant increase in the coefficient of variation only in the *cifA*/*β-Spec* assay, which encompassed a broader concentration range (Pearson’s *R²* = 0.655, *P* = 0.0274, *N =* 7; Fig. 6E). These data confirm that the *cif*/*β-Spec* assays are precise, with a predictable decline in precision when counting low-abundance targets.

## DISCUSSION

CI strength directly influences *Wolbachia* prevalence in natural and applied insect populations (6, 20), and stronger CI often correlates with higher *cifB* transcript levels (24). The naturally low abundance of *cifB* transcripts (34), however, necessitates the pooling of tissues from 15 or more individuals to achieve reliable quantification through standard RT-qPCR procedures. Here, we validate a phase-separation RNA extraction procedure that is equally efficient from 1 to 20 pairs of testes, demonstrate that genomic DNA can be completely removed through DNase treatment, and introduce four RT-ddPCR assays that reliably detect 1–3 *cifA* or *cifB* copies. Together, these findings establish that these assays are suitable to count rare *cifA* and *cifB* transcripts from low-biomass samples. Below, we discuss the appropriate application for each assay, strategies for further improving assay sensitivity and precision, and generalizability beyond *w*Mel in *D. melanogaster*. While these RT-ddPCR assays demonstrate detection of low-abundance *cif* transcripts from low-biomass samples, direct experimental comparison with RT-qPCR methods is required to quantitatively assess improvements in sensitivity.

To measure *cif*-gene expression, we developed two distinct assays for each *cif* gene, *cif*/spike and *cif*/*β-Spec*, which enable different normalization strategies. The *cif*/spike assays control for technical variability by introducing a synthetic spike-in RNA into each sample prior to RNA extraction. We assume that any loss of this spike-in RNA during downstream processing (e.g., purification, DNase treatment, cDNA synthesis) is proportional to the loss of endogenous *cif* transcripts. By measuring the recovery of the spike-in RNA via RT-ddPCR, we calculate processing efficiency for each sample. Multiplying raw *cif* counts against processing efficiency corrects for technical variation introduced during sample handling. In contrast, the *cif*/*β-Spec* assay normalizes *cif* transcript counts to those of an endogenous reference gene. Reference gene normalization is widely used to account for variation in starting material (e.g., tissue abundance, cell numbers) and transcriptional activity. This method assumes stable expression of the reference gene across conditions, enabling quantification of relative transcript abundance (46). We selected *β-Spec*, a single-copy *D. melanogaster* gene encoding a cytoskeletal protein essential for cell structure and membrane integrity, based on two key criteria. First, *β-Spec* is stably expressed throughout embryogenesis, pupal developmental stages, and as adult *D. melanogaster* age (47). Second, we have validated stable *β-Spec* expression through RT-qPCR in males of different ages that induce different CI strengths (27). However, the validity of reference gene normalization depends entirely on expression stability under the specific conditions being tested. Before using the *cif*/*β-Spec* assays, it is crucial to confirm that *β-Spec* expression remains stable across all experimental conditions. For experiments where host gene expression stability cannot be confirmed, the *cif*/spike assays provide a more robust alternative, as the exogenous spike-in control is independent of host physiology.

We defined the limit of detection for each assay as the upper limit of the 95% CI of the no-template controls (rounded up); this value establishes the threshold below which a signal is indistinguishable from background noise. We calculated limits of detection as one *cifA* copy for both assays, three *cifB* copies for the *cifB*/spike assay, and one *cifB* copy for the *cifB*/*β-Spec* assay. Since these values are lower than the transcript quantities measured in dilutions equivalent to individual testes pairs, we conclude that these assays are sufficiently sensitive to measure even rare *cif* transcripts from individual insect tissues. However, the limit of detection can vary between experiments depending on background signal levels, which are influenced by lab cleanliness and handling techniques. Therefore, we strongly recommend including both no-template and gDNA elimination controls in every run. Furthermore, while both assays are accurate and precise, precision is marginally lower when counting rare targets. This result is consistent with our prior measurements of *Wolbachia* abundance with ddPCR (42) and is an expected consequence of stochastic variation during pipetting with rare targets and low volumes. Although the assays are highly sensitive and precise, their performance can be further improved by increasing the number of molecules analyzed. This can be achieved by resuspending the RNA in a smaller volume than the current 25 μL, increasing the current 2 μL of cDNA per reaction to the maximum allowable volume of 7.5 μL for the *cif*/spike or 8 μL for the *cif*/*β-Spec* assays, and increasing the number of droplets analyzed by performing multiple RT-ddPCRs for each sample. Crucially, when using column-based RNA extraction kits, decreasing the elution volume may not yield a proportional increase in concentration and can result in sample loss, as insufficient elution volumes cannot effectively recover all RNA bound to the column matrix.

While the *cif*/*β-Spec* RT-ddPCR assays are specific to *D. melanogaster*, the *cif*/spike assays do not measure any host transcripts, making them host-independent. Therefore, we expect the *cif*/spike assays to be applicable to biocontrol programs that deploy *w*Mel *Wolbachia* in *Ae. aegypti* mosquitoes to control viral pathogens such as dengue and Zika (18). Since strong CI facilitates *w*Mel’s spread, and weak CI can lead to failed public health initiatives (48), these assays may enable efforts to monitor *cif* transcript levels in field-caught mosquitoes as a proxy for CI-strength variation. Furthermore, we designed the *cif* oligos with homology to at least 39 *cifA* variants (from 32 *Wolbachia* strains) and 34 *cifB* variants (from 27 *Wolbachia* strains). This extends the application of these assays to an array of *Wolbachia*-host systems, including agricultural pests like *Delia radicum* (49, 50), *Liriomyza huidobrensis* (51, 52), and *Rhagoletis cingulata* (53), which destroy crops, such as cabbage, peas, beans, and cherries; the livestock pest *Haematobia irritans* (54); and *Hofmannophila pseudospretella*, which damages stored cereals, fabrics, and dried fruit. Crucially, in this study, we have validated these assays for only *w*Mel. Applying these assays to any of the aforementioned systems should be preceded by pilot experiments to confirm their suitability.

In conclusion, we present accurate, precise, and sensitive RT-ddPCR assays to count *cifA* and *cifB* transcripts from low-biomass samples. This includes assays designed to control for technical variation in RNA processing and to normalize against an endogenous *D. melanogaster* reference gene. We expect these tools to accelerate research on CI by enabling studies with individual-level resolution of *cif* transcription in *w*Mel *Wolbachia* and, potentially, in up to 33 other *Wolbachia* strains. Individual-level measurements will enable pairing of *cif* transcript levels and CI strength from the same individual, facilitating studies of CI variation in complex laboratory and field conditions where inter-individual variation is high. These assays may be particularly valuable when paired with DNA-based ddPCR methods (42), enabling concurrent quantification of *Wolbachia* titer and *cif* expression from individual insects and detailed molecular characterization of the mechanisms underlying CI-strength variation (7). Crucially, this methodology can be easily adapted to quantify other low-abundance transcripts by altering the oligo sequences used in the RT-ddPCR, enabling quantification of divergent *cifs*, other *Wolbachia* effector genes (55), or any other target where individual-level resolution could be valuable.

## MATERIALS AND METHODS

### Insect lines, care, and maintenance

Experiments were performed using *w*Mel-bearing *D. melanogaster* from the y^1^w^*^ stock (BDSC 1495). Flies were maintained under a 12:12 light:dark cycle at 23°C within a *Drosophila* incubator (Percival DR-36VL) using standard narrow *Drosophila* vials (Flystuff 32-113RL) containing 7 mL to 10 mL of fly food (see reference 56 for detailed protocol). Adult flies were anesthetized with CO_2_ during experiments. *Wolbachia* cytotypes were periodically tested by extracting DNA from pools of three randomly sampled flies from each stock using a SquishBuffer method. This was followed by PCR amplification of the *Wolbachia* surface protein gene and the host 28S rDNA gene and gel electrophoresis (see reference 28 for detailed protocol).

### Sample collection, RNA extraction, and RNA processing

The following methods are detailed in full on protocols.io (57). To collect samples, males were anesthetized with CO_2_, their testes were dissected in 1× RNase-free PBS (Fisher Bioreagents, BP3994), and tissue was transferred to 2 mL centrifuge tubes (Eppendorf, 05414203) containing 800 µL of chilled TRIzol (Invitrogen, 15596026) and three 2.8 mm ceramic homogenizing beads (VWR, 10158-554). Males were randomly collected from experimental working stocks; therefore, age, mating history, developmental timing, paternal grandmother age, and other physiological factors known to impact CI strength were not controlled. Since CI is sensitive to host physiological parameters, this approach likely sampled males with variable *cif* expression, including suboptimal levels, thereby challenging the assay’s detection limits. The samples were immediately homogenized in a bead-mill homogenizer at 1,500 rpm for 2 min (Benchmark Scientific, BeadBlaster 96), centrifuged to bring the contents to the bottom of the tube, and frozen at −80°C until processing.

RNA was extracted using TRIzol:chloroform phase separation. Samples were initially thawed, homogenized again for 2 min at 1,500 rpm in a bead mill, and incubated for 5 min at room temperature. A synthetic spike-in RNA sequence (TATAA Biocenter, RS25SI) was diluted 1:64, and 1 µL was added to each sample. After 160 µL of chloroform (Thermo Fisher Scientific, 032614.K2) was added, samples were incubated for 5 min at room temperature, centrifuged at 4°C for 15 min at 12,000 × *g*, and the upper aqueous phase was collected. Three microliters of glycogen (20 µg/µL; Invitrogen, 10814010) and 400 µL of isopropanol (Thermo Fisher Scientific, 327272500) were added to each sample. Samples were incubated for 10 min at room temperature and centrifuged at 4°C for 20 min at 12,000 × *g*. The supernatant was then discarded, and the pellet was washed four times with 500 µL of 75% ethanol (Koptec, V1001), with intermittent 5-min centrifugations being performed at 4°C and 7,500 × *g*. After the RNA pellet was air-dried, the RNA was dissolved in 25 µL of low EDTA TE buffer (Quality Biological, 351-324-721). Reprecipitation was performed to further clean the sample by adding 62.5 µL of 200 proof ethanol and 2.5 µL of 3 M NaAc (pH 5.2) to each sample, and samples were stored overnight at −20°C. Following a 30-minute centrifugation at 4°C and 21,000 × *g*, the pellets were washed three times with ice-cold 75% ethanol, as described above. After the RNA pellet was air-dried, the RNA was dissolved in 25 µL of low EDTA TE. A NanoDrop 1C Spectrophotometer (Thermo Fisher Scientific, ND-ONE-W) was used to evaluate the quality of each sample, and a Qubit 4 Fluorometer (Thermo Fisher Scientific, Q33238) with RNA High Sensitivity (HS) Assay Kit (Invitrogen, Q32855) was used to measure RNA quantity.

For the DNA treatment experiment, DNase treatment was performed using three protocols from the DNA-free kit (Invitrogen, AM1906) and two protocols from the TURBO DNase kit (Invitrogen, AM1907). The manufacturer’s recommendations were followed for each protocol. For the DNA-free kit, the “routine” and “rigorous” protocols were carried out, and the rigorous protocol was also performed twice. For the TURBO DNase kit, both the “routine” and “rigorous” protocols were performed. For both kits, the routine protocol involved adding 1 µL of DNase and 2 µL DNase buffer to 20 µL of RNA, incubating at 37°C for 30 min, adding an inactivation reagent, and transferring the supernatant after pelleting the inactivation reagent. The rigorous protocol differed only in that another 1 µL of DNase was added after the initial 30-minute incubation, and incubation was continued for an additional 30 min. Whether DNA was removed from the reaction was initially determined by performing PCR for the 28S rDNA of *D. melanogaster* and gel electrophoresis (see reference 28 for detailed protocol). First-strand cDNA synthesis was performed using the SuperScript IV VILO Master Mix (Invitrogen, 11756050). cDNA was either immediately used for RT-ddPCR or stored at −80°C. The routine TURBO DNase protocol was used for all other experiments.

### Oligo design for ddPCR

Three new RT-ddPCR oligo sets (primers and probes) were designed for this study, targeting *cifA*, *cifB*, and *β-Spec* (FlyBase ID FBgn0250788; Table 1). To design the *cif* oligo sets, relevant sequences were retrieved from an in-house collection of *Wolbachia* genomes using BLAST (e-value 1e−10) with *w*Mel *cif* sequences as queries. A multiple sequence alignment (MSA) was generated using Muscle5 (58), and a consensus sequence was obtained with variable sites represented as Ns using Geneious Prime v2024.0.5. Primer3web v4.1.0 (59) was used to design oligos based on the consensus sequence. The sequence list was iteratively refined through multiple rounds of MSA and oligo design until oligos matching the criteria below were acquired. Primer-BLAST was used to determine the number of *cif* variants and *Wolbachia* strains that the *cif* oligos are homologous to in the NCBI nr database. The *β-Spec* oligo set was designed using Primer3web based solely on the *D. melanogaster* sequence (GenBank M92288).

**TABLE 1:** RT-ddPCR oligos for measuring *cifA* and *cifB* mRNA

Target	Forward (5′−3′)	Reverse (5′−3′)	Probe (5′−3′)	Amplicon size (bp)
cifAwMelT1_dd	GGTCCTTGGAATAATTTGCGG	TCAAACTCAGACTGTGGGC	TTGCCACTTGATGGTTCTGGTGA	83
cifBwMelT1_dd	GCAAGGTACTAGAGCACAGG	CACGAGCGTTGTTTCTACG	AGGTGGTACTTCTACAGCACAAGG	122
β-Spec_dd	ATGACGACGGACATTTCGATTG	AACAGTCGGGAACTGGAGTTG	GGTCCTGGCAACGAGTACATCGAT	107

Primers were between 18 and 22 base pairs (bp) long, and amplicons ranging from 70 to 150 bp were yielded. They also had a GC content of 40 to 60%, a GC clamp of 2, a melting temperature (Tm) of 58 to 62°C, a maximum Tm difference of 2°C, and a maximum of three consecutive identical nucleotides (poly-X). Probes were 18 to 30 bp in length with a Tm of 65 to 70°C, a GC content of 30 to 80%, and a maximum of 5 consecutive identical nucleotides. Tm calculations were performed using the Santa Lucia algorithm (60) with specified ion and dNTP concentrations. To ensure specificity, primers were analyzed against human (taxid: 9606), *D. melanogaster* (taxid: 7227), and *Wolbachia* (taxid: 953) genomes using Primer-BLAST. Premixed primer-probe sets were purchased from Bio-Rad at a 900 nM:250 nM ratio. Proprietary oligos for the synthetic spike-in RNA were purchased from TATAA Biocenter (RS25SI).

### *Wolbachia* phylogeny

*Wolbachia* genomes used in this study are listed in Table S1. Phylogenetic reconstruction was performed by first identifying orthologs of known bacterial genes using Prokka (v.1.14.5) (61). Genes present in single copy in each genome were extracted and aligned with MAFFT (v.7) (62). To avoid frameshifts and pseudogenes, genes with alignment gaps not in multiples of three or where more than one sample had gaps were dropped. A total of 149 genes (117,282 base pairs) met these criteria. Phylogenetic inference was conducted using RevBayes (v.1.1.1) (63), following previously established methods (24, 42, 64).

### RT-ddPCR

All RT-ddPCRs were conducted in 20 µL volumes. *cifA*/spike and *cifB*/spike duplex reactions were prepared using 10 µL of ddPCR Supermix for Probes (no dUTP) (Bio-Rad, 1863023), 1 µL of the relevant *cif* oligo set, 1 µL spike-in RNA primer mixture, 0.5 µL spike-in RNA probe, 5.5 µL of nuclease-free water, and 2 µL of cDNA template. *cifA*/*β-Spec* and *cifB*/*β-Spec* duplex reactions were prepared using 10 µL of ddPCR Supermix for Probes (no dUTP) (Bio-Rad, 1863023), 1 µL of each relevant oligo set, 6 µL of nuclease-free water, and 2 µL of cDNA template. After preparation, PCR plates were sealed with an adhesive film (Bio-Rad, MSB1001), vortexed for 10 s to mix (Four E’s Scientific, MI0101002), and centrifuged for 2 min at 2,204 × *g* (Eppendorf, 5430-R). The adhesive film was then removed, and 19.5 µL of each reaction mixture was transferred to a droplet generation cartridge (Bio-Rad, 1864007). Seventy microliters of droplet generation oil for probes (Bio-Rad, 1863005) was added to the adjacent well, a gasket was placed (Bio-Rad, 1864007), and the cartridge was positioned in a droplet generator (Bio-Rad, QX200) to create droplets. Next, the 40 µL droplet wells were transferred to a 96-well plate, sealed with a heat-activated adhesive film (Bio-Rad, 1814040 and PX1), and placed in a thermal cycler (Bio-Rad, C1000 or S1000) for PCR. Reaction conditions varied across experiments, as described in Results. After PCR, samples were maintained at 12°C until analysis on a droplet reader (Bio-Rad, QX200). Samples that yielded fewer than 10,000 droplets were excluded. A detailed RT-ddPCR protocol for *cif* transcript analysis is presented on protocols.io (65).

### Statistical analysis and figure generation

QX Manager software (v.2.1.0; Bio-Rad) was used to calculate RT-ddPCR target concentration and confidence intervals and to produce RT-ddPCR plots. All other statistical analyses were performed in R (v.4.4.1) using RStudio (v2024.04.2). To create plots, the "ggplot2" package (v.3.4.4) was used in R (66, 67). Finally, Inkscape (v.1.3.2; Inkscape Developers) was used to modify figure aesthetics.

## Data Availability

All data are publicly available at https://datadryad.org/dataset/doi:10.5061/dryad.0k6djhbd8.
